# In Vitro Wound Healing Improvement by Low-Level Laser Therapy Application in Cultured Gingival Fibroblasts

**DOI:** 10.1155/2012/719452

**Published:** 2012-07-15

**Authors:** Fernanda G. Basso, Taisa N. Pansani, Ana Paula S. Turrioni, Vanderlei S. Bagnato, Josimeri Hebling, Carlos A. de Souza Costa

**Affiliations:** ^1^Faculdade de Odontologia de Piracicaba, Universidade Estadual de Campinas (UNICAMP), 13414-903 Piracicaba, SP, Brazil; ^2^Faculdade de Odontologia de Araraquara, Universidade de Estadual Paulista (UNESP), 14801-903 Araraquara, SP, Brazil; ^3^Instituto de Física de São Carlos, Universidade de São Paulo (USP), 13560-970 São Carlos, SP, Brazil; ^4^Departamento de Fisiologia e Patologia, Faculdade de Odontologia de Araraquara, Universidade Estadual Paulista, Rua Humaitá, 1680, Centro, Caixa Postal: 331, 14801903 Araraquara, SP, Brazil

## Abstract

The aim of this study was to determine adequate energy doses using specific parameters of LLLT to produce biostimulatory effects on human gingival fibroblast culture. Cells (3 × 10^4^ cells/cm^2^) were seeded on 24-well acrylic plates using plain DMEM supplemented with 10% fetal bovine serum. After 48-hour incubation with 5% CO_2_ at 37°C, cells were irradiated with a InGaAsP diode laser prototype (LASERTable; 780 ± 3 nm; 40 mW) with energy doses of 0.5, 1.5, 3, 5, and 7 J/cm^2^. Cells were irradiated every 24 h totalizing 3 applications. Twenty-four hours after the last irradiation, cell metabolism was evaluated by the MTT assay and the two most effective doses (0.5 and 3 J/cm^2^) were selected to evaluate the cell number (trypan blue assay) and the cell migration capacity (wound healing assay; transwell migration assay). Data were analyzed by the Kruskal-Wallis and Mann-Whitney nonparametric tests with statistical significance of 5%. Irradiation of the fibroblasts with 0.5 and 3 J/cm^2^ resulted in significant increase in cell metabolism compared with the nonrradiated group (*P* < 0.05). Both energy doses promoted significant increase in the cell number as well as in cell migration (*P* < 0.05). These results demonstrate that, under the tested conditions, LLLT promoted biostimulation of fibroblasts in vitro.

## 1. Introduction

Tissue healing involves an intense activity of diverse cell types, such as epithelial and endothelial cells, as well as fibroblasts which play a key role in this process [1]. Fibroblasts secrete multiple growth factors during wound reepitelialization and participate actively in the formation of granulation tissue and the synthesis of a complex extracellular matrix after reepitelialization [1]. All these processes directly involve the proliferation and migration capacity to these cells [1]. The use of low-level laser therapy (LLLT) has been proposed to promote biostimulation of fibroblasts and accelerate the healing process [2].

Previous studies have evaluated the effect of LLLT on the proliferation and migration of human gingival fibroblasts as well as other cellular effects and responses, such as protein production and growth factor expression [2–6]. Nevertheless, there is a shortage of studies investigating irradiation parameters capable of promoting biostimulatory effects on fibroblasts in order to establish an ideal irradiation protocol for these cells [7]. Therefore, the aim of this study was to determine the most adequate energy doses using specific parameters of LLLT to produce biostimulatory effects on human gingival fibroblast cultures in an in vitro wound healing model. 

## 2. Material and Methods

### 2.1. Gingival Fibroblast Cell Culture

All experiments were performed using human gingival fibroblast cell culture (continuous cell line; Ethics Committee 64/99-Piracicaba Dental School, UNICAMP, Brazil). The fibroblast cells were cultured in Dulbecco's Modified Eagle's Medium (DMEM; Sigma-Aldrich, St. Louis, MO, USA) supplemented with 10% fetal bovine serum (FBS; Gibco, Grand Island, NY, USA), with 100 IU/mL penicillin, 100 *μ*g/mL streptomycin, and 2 mmol/L glutamine (Gibco, Grand Island, NY, USA) in an humidified incubator with 5% CO_2_ and 95% air at 37°C (Isotemp; Fisher Scientific, Pittsburgh, PA, USA) [8]. The cells were subcultured every 2 days in the incubator under the conditions described above until an adequate number of cells were obtained for the study. The cells (3×10^4^ cells/cm²) were then seeded on sterile 24-well acrylic plates using plain DMEM supplemented with 10% FBS for 48 h.

### 2.2. LLLT on Fibroblast Culture

The LLLT device used in this study was a near infrared indium gallium arsenide phosphide (InGaAsP) diode laser prototype (LASERTable; 780 ± 3 nm wavelength, 0.04 W maximum power output), which was specifically designed to provide a uniform irradiation of each well (2 cm²) in which cultured cells are seeded [8, 9]. The power loss through the acrylic plate was calculated using a potentiometer (Coherent LM-2 VIS High-Sensitivity Optical Sensor, USA), which was placed inside the culture plate. After this measure, the power loss of the plate was determined as 5%. After that, the power of all diodes was checked and standardized. Therefore, a final power of 0.025 W reached the cultured cells. This standardization was performed as previously described in the literature [8, 9]. For the evaluation of cell metabolism, the radiation originated from the LASERTable was delivered on the base of each 24-well plate with energy doses of 0.5, 1.5, 3, 5, and 7 J/cm², and irradiation times of 40, 120, 240, 400, and 560 s, respectively. The laser light reached the cells on the bottom of each well with a final power of 0.025 W because of the loss of optical power in each well due to the interposition of the acrylic plate. The cells were irradiated every 24 h totalizing 3 applications during 3 consecutive days. The cells assigned to control groups received the same treatment as that of the experimental groups. The 24-well plates containing the control cells were maintained at the LASERTable for the same irradiation times used in the respective irradiated groups, though without activating the laser source (sham irradiation) [8, 9]. Twenty-four hours after the last irradiation (active or sham), the metabolic activity of the cells was evaluated using the MTT assay (described below). Based on cell metabolism results, the two most effective irradiation doses were selected to evaluate the cell number (trypan blue assay), cell migration capacity by using the wound healing assay (qualitative analysis) and the transwell migration assay (quantitative analysis), as described below.

### 2.3. Analysis of Cell Metabolism (MTT Assay)

Cell metabolism was evaluated using the methyltetrazolium (MTT) assay [8–10]. This method determines the activity of succinic dehydrogenase (SDH) enzyme, which is a measure of cellular (mitochondrial) respiration and can be considered as the metabolic rate of cells.

Each well with the fibroblasts received 900 *μ*L of DMEM plus 100 *μ*L of MTT solution (5 mg/mL sterile PBS). The cells were incubated at 37°C for 4 h. Thereafter, the culture medium (DMEM; Sigma Chemical Co., St. Louis, MO, USA) with the MTT solution were aspirated and replaced by 700 *μ*L of acidified isopropanol solution (0.04 N HCl) in each well to dissolve the violet formazan crystals resulting from the cleavage of the MTT salt ring by the SDH enzyme present in the mitochondria of viable cells, producing a homogenous bluish solution. Three 100 *μ*L aliquots of each well were transferred to a 96-well plate (Costar Corp., Cambridge, MA, USA). Cell metabolism was evaluated by spectrophotometry as being proportional to the absorbance measured at 570 nm wavelength with an ELISA plate reader (Thermo Plate, Nanshan District, Shenzhen, China) [8, 9]. The values obtained from the three aliquots were averaged to provide a single value. The absorbance was expressed in numerical values, which were subjected to statistical analysis to determine the effect of LLLT on the mitochondrial activity of the cells. 

### 2.4. Viable Cell Counting (Trypan Blue Assay)

Trypan blue assay was used to evaluate the number of cells in the culture after LLLT application. This test provides a direct assessment of the total number of viable cells in the samples as the trypan blue dye can penetrate only porous, permeable membranes of lethally damaged (dead) cells, which is clearly detectable under optical microscopy [11]. The LLLT protocol was undertaken as previously described using energy doses of 0.5 and 3 J/cm². Cell counting was performed in the experimental and control groups 24 h after the last irradiation (active or sham). The DMEM in contact with the cells was aspirated and replaced by 0.12% trypsin (Invitrogen, Carlsbad, CA, USA), which remained in contact with the cells for 10 min to promote their detachment from the acrylic substrate. Then, 50 *μ*L aliquots of this cell suspension were added to 50 *μ*L of 0.04% trypan blue dye (Sigma Aldrich Corp., St. Louis, MO, USA), and the resulting solution was maintained at room temperature for 2 min so that the trypan blue dye could pass through the cytoplasmic membrane of the nonviable cells, changing their color into blue. Ten microliters of the solution were taken to a hemocytometer and examined with an inverted light microscope (Nikon Eclipse TS 100, Nikon Corporation, Tokyo, Japan) to determine the number of total cells and nonviable cells. The number of viable cells was calculated by deducting the number of nonviable cells from the number of total cells [8]. The number of cells obtained in the counting corresponded to *n* × 10^4^ cells per milliliter of suspension.

### 2.5. Cell Migration

#### 2.5.1. Wound Healing Assay

The wound healing assay was used because it is a classic method of evaluation in vitro tissue healing assays [12, 13]. After 48 h of cell culture, a sterile 5 mL pipette tip was used to make a straight scratch on the monolayer of cells attached to the acrylic substrate, simulating a wound. Formation of the in vitro wound was confirmed under an inverted microscope (TS 100, Nikon, Tokyo, Japan). The LLLT protocol was undertaken as previously described using energy doses of 0.5 and 3 J/cm². Twenty-four hours after the last irradiation, the cells were fixed in 1.5% glutaraldehyde for 1 h, stained with 0.1% violet crystal for 15 min, and washed twice with distilled water. Wound repopulation was assessed with a light microscope (Olympus BX51, Miami, FL, USA) equipped with a digital camera (Olympus C5060, Miami, FL, USA).

#### 2.5.2. Transwell Migration Assay

The capacity of human gingival fibroblasts to migrate through a cell permeable membrane was assessed using 6.5 mm-diameter transwell chambers (Corning Costar, Cambridge, MA, USA) with polycarbonate membrane inserts (8 *μ*m pore size) [14]. The chambers were placed in 24-well plates containing 1 mL of plain DMEM per well. The cells were seeded onto the upper compartment of the chamber (1.5 × 10^4^ cells/cm²) and incubated at 37°C for 48 h. After this period, the LLLT protocol was undertaken as previously described using energy doses of 0.5 and 3 J/cm². Twenty-four hours after the last irradiation (active or sham), the cells that had migrated through the membrane to the lower compartment of the chamber were fixed in 1.5% glutaraldehyde for 1 h, incubated with 0.1% violet crystal dye for 15 min, and washed twice with distilled water. After the last wash, the stained cells were viewed under a light microscope (Olympus BX51, Miami, FL, USA) equipped with a digital camera (Olympus C5060, Miami, FL, USA) and photomicrographs from three randomly chosen fields were taken at ×10 magnification for counting the number of migrated cells using the image-analysis J 1.45S software (Wayne Rasband, National Institutes of Health, Bethesda, MD, USA). Two samples of each group were evaluated and the experiment was performed in triplicate. 

### 2.6. Analysis of Migrated Cells by Scanning Electron Microscopy (SEM)

Part of the specimens used in the transwell migration assay was also used for the analysis of the cells by SEM. Twenty-four hours after the last irradiation (active or sham), the culture medium was aspirated and the transwell inserts were fixed in 1 mL of 2.5% glutaraldehyde in PBS for 2 h. Then, the glutaraldehyde solution was aspirated and the cells adhered to the transwell inserts were washed with PBS and distilled water two consecutive times (5 min each) and then dehydrated in a series of increasing ethanol concentrations (30, 50 and 70%, one time for 30 min each; 95 and 100%, two times for 60 min each) and covered 3 times with  200 *μ*L of 1,1,1,3,3,3-hexamethyldisilazane (HMDS; Sigma Aldrich Corp., St. Louis, USA) [8]. The transwell inserts were stored in a desiccator for 24 h, sputter-coated with gold, and the morphology of the surface-adhered cells was examined with a scanning electron microscope (JMS-T33A scanning microscope, JEOL, Tokyo, Japan). 

### 2.7. Statistical Analysis

Data from MTT, Trypan blue and Transwell assay had a nonnormal distribution (Kolmogorov-Smirnov, *P* < 0.05) and were analyzed by the Kruskal-Wallis and Mann-Whitney nonparametric tests. A significance level of 5% was set for all analyses.

## 3. Results

### 3.1. Analysis of Cell Metabolism (MTT Assay)

Data from SDH production by human gingival fibroblast cultures (MTT assay) after LLLT, according to the energy dose are presented in Table 1.

Regarding the energy dose of 5 J/cm² no statistically significant difference between the irradiated group and the nonirradiated control group was observed (*P *> 0.05). Conversely, irradiation of the fibroblast cultures with doses of 0.5 J/cm² and 3 J/cm² resulted in 11% and 17% increases in cell metabolism, respectively, differing significantly from the control group (*P* < 0.05). The cells irradiated with 1.5 J/cm² and 7 J/cm² presented the lowest metabolic rate compared with the nonirradiated control group (6% and 8% decrease, resp., *P* < 0.05).

### 3.2. Viable Cell Counting (Trypan Blue Assay)

The number of viable cells (%) after LLLT application, according to the energy dose, is presented in Table 2.

Comparison among the energy doses revealed that irradiation of the human gingival fibroblast cultures with 0.5 J/cm² and 3 J/cm² increased the number of viable cells by 31% and 66%, respectively, differing significantly from the control (*P* < 0.05), but without statistically significant difference between each other (*P* > 0.05).

### 3.3. Fibroblast Migration

#### 3.3.1. Wound Healing Assay

**Figure 1:**
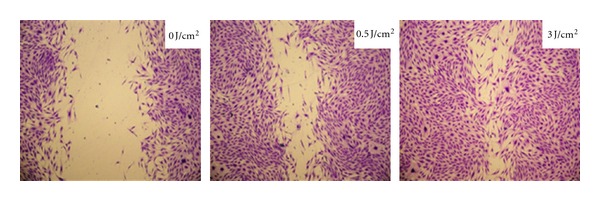
Photomicrographs showing human gingival fibroblast cultures seeded in 24-well plates after LLLT. The control group exhibits a large cell-free area on acrylic surface. The group irradiated with 0.5 J/cm² exhibits cell proliferation and migration, with consequent reduction of the “in vitro wound” size. The group irradiated with 3.0 J/cm² presented more intense cell proliferation and migration, resulting in almost complete closure of the “in vitro wound.”

The analysis of the monolayer of human gingival fibroblasts after irradiation of the “in vitro wound” showed more intense cell migration, with consequent better coverage of the substrate (wound repopulation) (Figure 1).

#### 3.3.2. Transwell Assay

Data from the transwell assay after LLLT, according to the energy dose are, presented in Table 3. 

Comparison among the energy doses revealed that irradiation of the human gingival fibroblast cultures with 0.5 J/cm² and 3 J/cm² increased cell migration by 16% and 18%, respectively, differing significantly from the control (*P* < 0.05), but without statistically significant difference between each other (*P* > 0.05). 

### 3.4. Analysis of Migrated Cells by Scanning Electron Microscopy (SEM)

**Figure 2:**
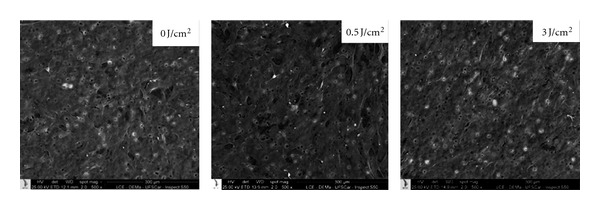
SEM micrograph showing cells with normal morphology that migrated through the transwell membrane. SEM ×500.

The SEM analysis of the transwell inserts, which complemented the viable cell counting by the trypan blue assay, revealed that the fibroblasts were capable of migrating through the transwell membrane. The cells obtained from human gengiva did not change their morphology after been submitted to LLLT (Figure 2).

## 4. Discussion

Different LLLT modalities have been used for diverse treatments in the health fields. In Dentistry, LLLT has been widely investigated and indicated for accelerating the healing process, especially in the treatment of ulcerative oral mucosa lesions [15, 16].

Several in vitro studies have evaluated the effect of LLLT on healing [7, 17]. Nevertheless, current research involving irradiation of cell cultures has not yet established the irradiation patterns specific for the different cell lines. Establishing the ideal irradiation parameters and techniques is mandatory for the development of sequential studies that can determine the potential biostimulatory effect of LLLT on oral mucosa cells, such as keratinocytes and fibroblasts, which are directly involved in the local healing process.

In the present study, the metabolic activity of human gingival fibroblast cultures after LLLT with different energy doses was evaluated to determine the adequate doses to produce biostimulatory effects on these cells in vitro. The results for SDH production showed that the 0.5 and 3 J/cm² doses increased cell metabolism. Therefore, these two most effective irradiation doses were selected to evaluate the number of viable cells as well as the cell migration capacity. The increase of SDH production after irradiation of gingival fibroblasts has also been observed by Damante et al. [18], using a similar laser prototype to the one used in the present study. In the same way as in the present study, the SDH production results also served as guide for subsequent experiments that evaluated the expression of growth factors by cultured fibroblasts.

In the present study, a significant increase in the number of viable cells that presented normal morphological characteristics (SEM analysis) was observed after LLLT using doses of 0.5 and 3 J/cm^2^. These results confirm those of previous laboratory investigations in which LLLT with the same wavelength as that of the present study (780 nm) increased the proliferation of gingival fibroblasts [19, 20]. Kreisler et al. [2] also reported increase of fibroblast cell culture in vitro after direct and consecutive low level laser irradiations. The mechanism by which LLLT can promote biostimulation and induce proliferation of different cell types remains a controversial subject [20, 21]. Some authors [21, 22] claim that this mechanism is derived from light absorption by the enzyme cytochrome c oxidase in the cells, which participates in the cascade of oxidative respiration. Eells et al. [23] demonstrated the increase in the production of this enzyme after different LLLT application of cell cultures. It has also been suggested that the mechanism of cell proliferation induced by LLLT might be derived from the activation of singling pathways, such as the MAPK and PI3K/Akt pathways, which control both cell proliferation and regulation of gene expression [21, 24].

Fibroblast cell migration and proliferation are essential events for tissue healing and are directly related with its success [1, 3]. In the present study, the effect of LLLT on the capacity of gingival fibroblast migration, using two energy doses capable of increasing cell metabolism (0.5 and 3 J/cm²), was evaluated qualitatively, by the wound healing assay, and quantitatively, by the transwell migration assay. Both methodologies demonstrated that LLLT was able to increase the migration capacity of fibroblasts and the quantitative analysis of the results revealed no significant difference between the energy doses. These results are in accordance with those of previous investigations [7, 17], but studies using the transwell migration method to evaluate the LLLT on cell cultures are still scarce. This methodology is relevant because it measures the number of cells that can pass through the transwell membrane inserts, demonstrating their migration capacity after stimulation by LLLT.

Diverse mechanisms are involved in cell migration during tissue healing, including expression and secretion of growth factors [1]. Previous studies demonstrated that LLLT may cause positive effects on cells by increasing growth factor expression, which could be a form of action of specific laser parameters on cell migration [2, 25]. A recent study of our research group demonstrated that LLLT had a biostimulatory effect on epithelial cells in vitro by increasing their metabolic activity, number of viable cells and expression of growth factors [8]. In the present paper, the biostimulation of human gingival fibroblast cultures by LLLT with consequent increase in the number of viable cells and cell migration capacity demonstrates the efficacy of specific laser parameters and irradiation technique on the healing process. In addition, the obtained results are supportive to those of previous in vivo studies in which acceleration of the healing process was observed after LLLT [15, 16, 26], but the limitations of an in vitro experiment should be considered. 

In conclusion, the findings of the present study demonstrated that the preset laser parameters in combination with the sequential irradiation technique caused biostimulation, proliferation, and migration of human gingival fibroblast cultures. These encouraging laboratory outcomes should guide forthcoming studies involving tissue irradiation with laser and its effects on in vivo tissue healing.

## Figures and Tables

**Table 1:** Succinate dehydrogenase enzyme (SDH) production by human gingival fibroblasts detected by the MTT assay according to the energy dose used in the low-level laser therapy.

Energy dose (J/cm^2^)	MTT (%)
0 (control)	100 (96–104) C^∗^
0.5	111 (110–113) B
1.5	94 (92–97) D
3	117 (113–119) A
5	95 (81–108) CD
7	92 (91–96) D

Values expressed as medians of SDH production (P25–P75) (*n* = 12). ^∗^Same letters indicate no statistically significant difference (Mann-Whitney, *P* > 0.05).

**Table 2:** Number of viable cells (%) detected by the trypan blue assay, according to the energy doses used in the low-level laser therapy.

Energy dose (J/cm^2^)	Number of viable cells (%)
0 (control)	100 (95–104) B^∗^
0.5	133 (112–175) A
3	168 (149–181) A

Values expressed as medians of SDH production (P25–P75) (*n* = 8). ^∗^Same letters indicate no statistically significant difference (Mann-Whitney, *P* > 0.05).

**Table 3:** Cell migration (%) by the transwell assay, according to the energy dose used in the low-level laser therapy.

Energy dose (J/cm^2^)	Cell migration (%)
0 (control)	100 (91–107) B^∗^
0.5	118 (109–123) A
3	120 (116–122) A

Values expressed as medians of SDH production (P25–P75) (*n* = 6). ^∗^Same letters indicate no statistically significant difference (Mann-Whitney, *P* > 0.05).
